# Free and Immobilized Cells of *Torulaspora delbrueckii* and *Lachancea thermotolerans* in Sparkling Wine: Innovative Application in Secondary Bottle Fermentation

**DOI:** 10.3390/foods14173007

**Published:** 2025-08-28

**Authors:** Laura Canonico, Laura Moretti, Alice Agarbati, Francesca Comitini, Maurizio Ciani

**Affiliations:** Department of Life and Environmental Sciences, Polytechnic University of Marche, Via Brecce Bianche, 60131 Ancona, Italy; l.canonico@univpm.it (L.C.); laura.moretti@pm.univpm.it (L.M.); a.agarbati@univpm.it (A.A.); f.comitini@univpm.it (F.C.)

**Keywords:** secondary bottle fermentation, cell immobilization, volatile profile, non-*Saccharomyces*, sensorial profile

## Abstract

Sparkling wine production involves secondary alcoholic fermentation, during which carbon dioxide is trapped, creating effervescence and enhancing sensory complexity. This study evaluated the impact of *Torulaspora delbrueckii* and *Lachancea thermotolerans* yeast species using free and immobilized cells in secondary fermentation of sparkling wine, in comparison with *Saccharomyces cerevisiae*. Immobilized *S. cerevisiae* enabled faster refermentation compared to free cells, while immobilization resulted in a slower process in non-*Saccharomyces* strains. Biomass monitoring showed stable viable cells for immobilized *S. cerevisiae* during fermentation, while non-*Saccharomyces* strains showed a consistent reduction. Volatile profiles were positively influenced by immobilization using *S. cerevisiae* strains, which produced a constant increase in key aroma compounds, such as geraniol and ethyl acetate, throughout fermentation. Non-*Saccharomyces* strains contributed to enhanced fruity and floral aromas with variations in volatiles during refermentation. Sparkling wines fermented with immobilized *L. thermotolerans* were noted for ripe fruit aromas, while *T. delbrueckii* increased floral notes. *S. cerevisiae* fermentations showed higher acidity and balanced structure. These findings highlight the influence of yeast species and the yeast immobilization procedures in secondary fermentation, modulating fermentation dynamics and aroma development, and offer a promising strategy to tailor sparkling wine quality and sensory complexity.

## 1. Introduction

The Champenoise method of sparkling wine production is based on bottle fermentation of base wine after the addition of sugar and yeast. During *prise de mousse*, the yeast metabolism plays a relevant role, affecting the aroma and increasing the flavor of the final product [1]. The autolysis process, which takes place during the aging with yeast releasing intracellular yeast compounds, modifies the chemical composition and sensory properties of the wines. In this regard, the choice of the yeast species involved in the secondary fermentation in sparkling wine is crucial to obtaining a high flocculation capacity, good autolytic properties, and sensory properties of the wine [2,3,4,5,6]. The immobilization procedure consists of a confinement of yeasts cell on support which can be of different materials. The most used support for the immobilization procedure in winemaking is calcium alginate [6,7]. Calcium alginate has been approved by the International Organization of Vine and Wine (OIV) as a clarifying agent in the production of sparkling wine and as a matrix for the encapsulation of yeasts and bacteria (International Oenological Codex 2013) Today, immobilized *Saccharomyces cerevisiae bayanus* (Cremanti^®^, Erbslöh Cavis, Mainz, Germany) and *S. cerevisiae* (Prorestart^®^) are commercially available for the elaboration of sparkling wines using the Champenoise method [8].

Several studies explored the application of yeast immobilization—typically using calcium alginate, cellulose, or oak chips—as an alternative strategy during secondary fermentation in sparkling wine production [9] demonstrated that 2% alginate beads containing 10^9^ cells/g effectively prevent cell leakage and improve the organoleptic properties of the final product compared to conventional fermentation methods [10]. López de Lerma Extremera et al. [10] compared free, bio-immobilized, and alginate-immobilized yeasts after 32 months of bottle aging, observing that immobilized systems led to higher concentrations of key aroma compounds—such as isoamyl acetate and ethyl hexanoate—and clearer separation in PCA, indicating enhanced aromatic complexity. A more recent study [8] confirmed that immobilized yeasts do not significantly affect major oenological parameters (pH, acidity, effervescence) but increase free amino nitrogen while reducing total polysaccharides and proteins. Sensory analysis showed minor differences, mainly in the perception of “dough-like” aromas. The use of encapsulated *S. cerevisiae* was also investigated on the chemical and sensory profiles of sparkling cider produced by the Champenoise method [11].

Regarding the choice of yeast to be used in the production process of sparkling wines, generally strains belonging to the genus *Saccharomyces* are those that best adapt to the hostile conditions of bottle refermentation [12,13]. Recently, attention has also shifted to the application of non-*Saccharomyces* yeasts in sparkling wine production, given their important contribution in winemaking.

Several non-conventional yeasts were proposed for sparkling wine production with traditional methods. The most studied species of non-*Saccharomyces* yeasts for the production of sparkling wines have been *Torulaspora delbrueckii*, *Metschnikowia pulcherrima*, *Schizosaccharomyces pombe*, *Saccharomycodes ludwigii,* and *Starmerella bacillaris* [14,15,16]. Each of these yeast species contributes distinctively to sparkling wine. Specifically, *T. delbrueckii* has been shown to confer better foaming properties and improve the peculiar aromatic notes to give a distinctive attribute to emphasize the characteristics of sparkling wine. *S. ludwigii* and *S. pombe* contribute positively to organoleptic characteristics during the aging period on the lees of the sparkling wine. Moreover, *S. pombe* affected the sensorial profile, increasing the perception of some sensory notes such as floral, fruity, and buttery but also the structural aspects, including clarity and color intensity. Different fermentation strategies (pure and mixed fermentation) could be used to carry out the contribution of these non-*Saccharomyces* species to obtain specific sensory attributes.

In this study, the effects of *T. delbrueckii* and *Lachancea thermotolerans*, used in both free and immobilized forms, during the secondary fermentation of sparkling wine were evaluated and compared with *S. cerevisiae*. The impact of these fermentation strategies was assessed in terms of fermentation kinetics, volatile profile, and sensorial profile of the resulting sparkling wines.

## 2. Materials and Methods

### 2.1. Yeast Strains

The non-conventional yeasts used in this study were *T. delbuekii* DiSVA 130 and *L. thermotolerans* DiSVA 322. *S. cerevisiae* DiSVA 554 was used as a control strain. All strains were maintained in the Yeast Collection of the Department of Life and Environmental Sciences (DiSVA) of the Polytechnic University of Marche (Italy). For long-term storage at −80 °C, they were maintained in YPD broth on yeast extract (10 g/L), peptone (20 g/L), and dextrose (20 g/L) without agar supplemented with 40% (w/v) glycerol.

These yeasts were selected on the basis of a previous study that evaluated their fermentative performance and impact on the organoleptic properties of fermented beverages [14,17,18]. For short-term storage at 4 °C, the yeast strains were maintained.

### 2.2. Immobilization Procedure

The biomass for immobilized trials was obtained using a bench-top fermenter with modified YPD medium (0.5% yeast extract, 0.1% peptone, 2% dextrose, all w/v) sterilized at 121 °C for 20 min. *T. delbrueckii*, *L. thermotolerans*, and *S. cerevisiae* were inoculated in the media and incubated at 25 °C for 48 h in agitation (300 rpm). The biomass was harvested by centrifugation and mixed (10% wet w/v) with 2.5% Na-alginate at a ratio of 5% (wet w/v). The mixture was added dropwise into 0.1 M CaCl_2_ using a peristaltic pump to promote gelation. After 1 h, the resulting beads were rinsed multiple times with sterile distilled water and used immediately. The inoculum for the immobilized cells was 0.7% (wet w/v), which corresponded to an inoculum of ca. 10^6^ cells/mL.

### 2.3. Elaboration of Sparkling Wine

The base wine used for the elaboration of sparkling wines was a “Verdicchio” provided by Cantine Belisario, Matelica, MC, Italy. The main analytical characteristics of the base wine were total acidity (as tartaric acid), 6.57 g/L; volatile acidity, 0.25 g/L; pH, 3.42; ethanol, 10.92% vol.; residual sugar, 0.32 g/L; and total SO_2_, 63 mg/L. The strains were pre-cultured in 50 mL of modified YPD and incubated at 20 °C for 72 h. The biomass for free cells was harvested by centrifugation and inoculated in a flask containing 50 mL of white grape must, previously pasteurized, and 50 mL of sterile white wine with 20 mg/L DAP and Start Y Fresh (Station Oenoitechnique de Champagne Éperny Cedex France-) (10 mg/L), incubated in static condition at 20 °C for 9 days. After 3 days of inoculation, 150 mL of base wine was added to the flask incubated at 20 °C for 4–5 days.

After this period, 10^6^ cells/mL of yeast pre-culture were added to base wine supplemented with 24 g/L sucrose, 20 mg/L DAP, and 10 mg/L Start Y Fresh (Station Oenotechnique de Champagne, France). The mixture was then aliquoted into three 750 mL bottles for sparkling wine, sealed with crown caps and a so-called *bidule*.

### 2.4. Pressure and Biomass Evolution

The secondary fermentation was carried out at 19 °C, with the bottles stored horizontally while internal pressure was monitored using afrometers (Oenoitalia Group S.r.l., Erbusco (BS) Italy). Bottles were disgorged after 5 months. During the refermentation phase, bottles for each trial were collected and used to monitor the biomass evolution and volatile compounds during the *prise de mousse*. In the trials with the immobilized procedure, 1 g of beads containing the yeasts was collected and maintained under agitation in 50 mL of 1% Na-citrate solution (w/v) for 1 h to release the cells. The cell viability was evaluated by viable cell count (CFU/mL) on WL nutrient agar (Oxoid, Hampshire, UK).

### 2.5. Analytical Determination and the Quantification of the Main Volatile Compounds

The main analytical characters were determined at the end of refermentation using the standard OIV procedures. The methods used are the following: total acidity, volatile acidity, pH, ethanol content, and sugar content [19]. Acetaldehyde, ethyl acetate, and higher alcohol content were quantified with a gas chromatograph system (GC-2014; Shimadzu, Kyoto, Japan). The column used is a Zebron ZB-WAXPlus Phenomenex, Torrance, CA, USA (30 m × 0.32 mm column with a 0.25 μm film thickness) using 1-pentanol (162 mg/L) as an internal standard. The volatile compounds were determined with the solid-phase microextraction (HS-SPME) method extracted using a Divinylbenzene/Carboxen/Polydimethylsiloxane (DVB/CAR/PDMS) fiber (Sigma-Aldrich, St. Louis, MO, USA), following the procedure reported by [18]. The compounds were desorbed by inserting the fiber into a Shimadzu gas chromatograph (GC) injector for 5 min. The following glass capillary column was used: 0.25 μm Supelcowax 10 (length, 60 m; internal diameter, 0.32 mm). The fiber was inserted in split–splitless mode. The compounds were identified and quantified by comparisons with calibration curves for each compound. Acetaldehyde, ethyl acetate, higher alcohols, and the main volatile compounds were analyzed at different times of the refermentation phase and after 5 months.

### 2.6. Sensorial Analysis

A panel of ten qualified members (8 men and 2 women), aged from 25 to 60 years and experienced in the sensory evaluation of wine, was recruited to conduct a sensory evaluation of sparkling wines. The panel was composed of oenologists, sommeliers, and wine producers. Participation was voluntary, and all members provided informed consent; no compensation was offered. Subjects with known allergies or health problems were excluded from the study. Given the inherently safe nature of sparkling wines, ethical approval was not required. Sensory evaluations were conducted in a controlled environment to minimize external influences, at a constant temperature of 23 °C and illuminated with white light. Each taster was served 50 mL of sparkling wine in tasting glasses. Between tastings, drinking water was provided to cleanse the palate. Sensory evaluation was performed using a 1–10 scale (10 = maximum satisfaction). The panel was provided with a list of descriptors for olfactory and gustatory analysis. The data obtained were then statistically processed to evaluate the significant contribution that each fermentation procedure and each strain had given to the sparkling wine.

### 2.7. Statistical Analysis

Statistica 7, version 7, was used to evaluate the variance (ANOVA) of the main analytical characters and volatile compounds. Duncan’s test was used to determine the significant differences among the strains tested. The data were considered significant if the associated *p*-values were <0.05. The volatile compounds were also evaluated by the principal component analysis (PCA) to determine the eventual correlation among free and immobilized cells between the strains tested. PCA was performed in R-studio v2025.05.0 + 496(R Studio Team, 2012) with R 4.5.0. The data from the sensory analysis were also subjected to Fisher ANOVA to determine the significant differences (*p* < 0.05).

## 3. Results

### 3.1. Fermentation Kinetics and Biomass Evolution

**Figure 1:**
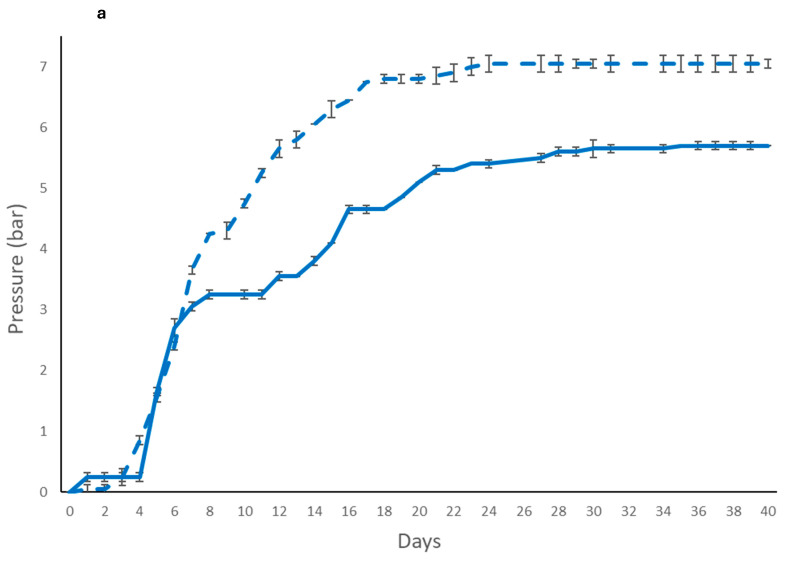

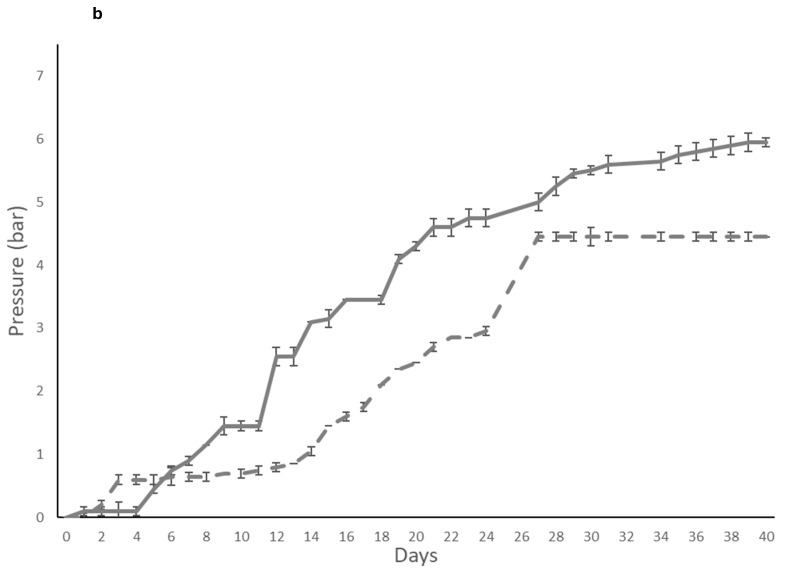

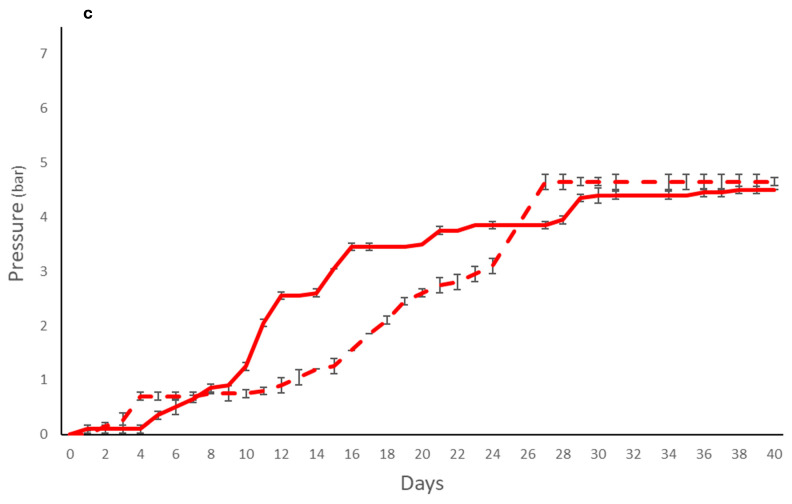
Fermentation kinetics of sparkling wines produced with free and immobilized cells. *S. cerevisiae* DiSVA 554: (**a**) free (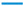) and immobilized (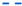); *T. delbrueckii* DiSVA 130: (**b**) free(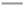) and immobilized (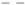); and *L. thermotolerans* DiSVA 322: (**c**) free (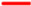) and immobilized (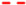).

The results of the fermentation kinetics were reported in Figure 1. *S. cerevisiae* free cells (Figure 1a) exhibited higher-pressure evolution in comparison with the two non-*Saccharomyces* strains tested. At the end of secondary fermentation *T. delbrueckii* (Figure 1b) achieved a comparable pressure to that exhibited by *S. cerevisiae* while *L. thermotolerans* (Figure 1c) showed a lower overpressure (4.5 bar). *S. cerevisiae* immobilized form (Figure 1a) showed a faster fermentation kinetics compared to the free cells, while *T. delbrueckii* (Figure 1b) immobilized cell showed a significant reduction in fermentation kinetics than free cells. *L. thermotolerans* (Figure 1c) exhibited the same trend in both conditions with a reduction on kinetics at middle of refermentation phase in comparison to free cells. Generally, the immobilization procedure negatively influenced the fermentation kinetic of the non-*Saccharomyces* yeasts. Differently, *S. cerevisiae* immobilized cells seem to increase the fermentation performance.

**Figure 2:**
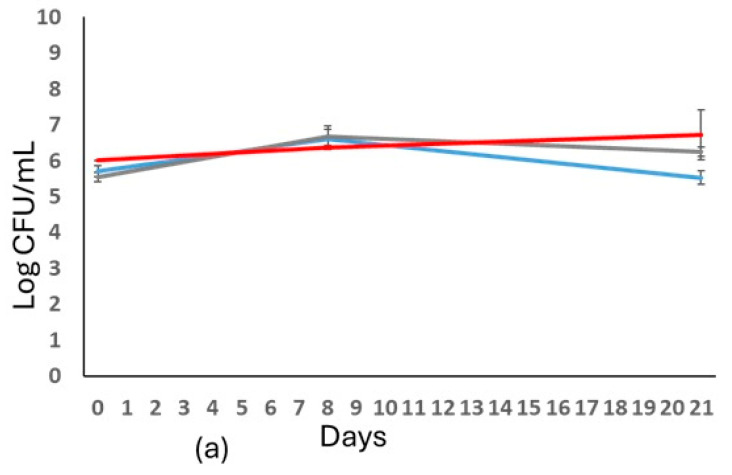

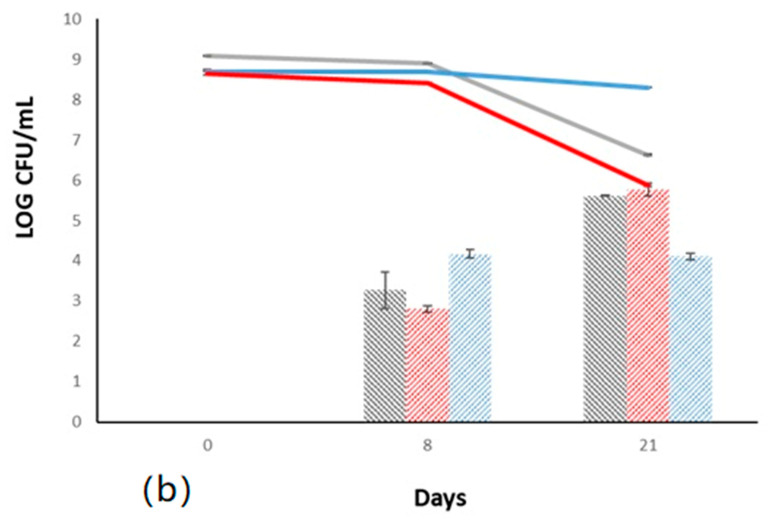
Biomass evolution of the strains in sparkling wine production with free (**a**) and immobilized cells (**b**). In (**a**): *S. cerevisiae* (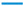); *T. delbrueckii* (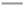); *L. thermotolerans* (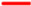). In (**b**): *S. cerevisiae* DiSVA 554 cell released (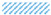); *T. delbrueckii* DiSVA 130 cell released (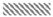); and *L. thermotolerans* DiSVA 322 cell released (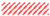). *S. cerevisiae *immobilized ( 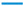); *T. delbrueckii* immobilized (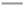); *L. thermotolerans* immobilized (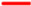).

The biomass evolution was evaluated during the refermentation process (Figure 2). The condition of free cells did not affect the evolution of growth kinetics, showing a similar trend among the strains tested. Regarding the immobilized conditions, the amount of *S. cerevisiae* immobilized remained constant during the process (days 8 and 21) with a cell release of 10^4^ cells/mL (Figure 2b). Conversely, the two non-*Saccharomyces* strains exhibited a reduction in immobilized viable cells at 21st. Regarding the cell release, both strains showed a similar trend: about 10^3^ cells/mL and 10^6^ cells/mL at days 8 and 21 of fermentation, respectively.

### 3.2. The Main Analytical Characters

The main analytical parameters of the sparkling wines of the two fermentation strategies (free and immobilized cells) are summarized in Table 1. A slight but statistically significant increase in total acidity content was observed in sparkling wines fermented with immobilized *S. cerevisiae*. Regarding the non-*Saccharomyces* strains, *T. delbrueckii* and *L. thermotolerans* immobilized cells led to a significant increase in residual sugar content. These differences may be attributed to strain-specific metabolic activity under immobilized conditions, which can impact sugar consumption efficiency and fermentation completeness. The use of both *T. delbrueckii* and *L. thermotolerans* in immobilized form led to a statistically significant increase in volatile acidity compared to the same strains in free form.

### 3.3. The Evolution of the Main Volatile Compounds

The main volatile compounds of sparkling wines were evaluated during the refermentation phase at different times (day 8, day 21, and the end of the refermentation) (Table 2 and Supplementary Materials).

The trends of the aroma compounds of *S. cerevisiae* varied considerably depending on both inoculation strategy (free vs. immobilized) and stage of the refermentation process. When *S. cerevisiae* was used in free-cell form, a statistically significant increase in the concentration of the major volatile compounds was observed during the early (day 8) and intermediate (day 21) stages of refermentation. This trend reflects an earlier phase of active metabolic activity during which the yeast contributes to the generation of aromatic complexity. However, at the end of the refermentation phase, most volatile compounds reach a plateau or even decrease slightly, with the notable exception of citronellol and phenyl ethyl acetate, which continue to increase significantly. Differently, *S. cerevisiae* used in immobilized form led to a more linear and progressive increase in volatile compound concentration throughout the entire refermentation process. Among volatile compounds, geraniol, ethyl acetate, and isobutanol showed significant and comparable increases at all time points. Furthermore, at the end of the refermentation phase, other volatile compounds, namely isoamyl acetate, hexanol, citronellol, and isoamyl alcohol, also showed statistically significant increases, indicating that immobilized yeast cells remained active in the subsequent stages of fermentation and contributed to further aroma enrichment.

On the 8th day of refermentation, *T. delbrueckii* in its free-cell form exhibited a significant increase in ethyl butyrate, isoamyl acetate, linalool, nerol, geraniol, β-damascenone, and isobutanol. These compounds are primarily associated with fruity and floral notes, indicating that *T. delbrueckii* actively contributes to the aromatic complexity of the wine in the early stages of secondary fermentation. By day 21, the volatile profile shifted, showing a significant accumulation of other compounds such as α-terpineol, phenyl ethyl acetate, β-phenyl ethanol, acetaldehyde, ethyl acetate, propanol, and isoamyl alcohol, suggesting a change in the metabolic activity of the yeast over time. At the end of the refermentation phase, only a limited number of volatile compounds, specifically hexanol, ethyl octanoate, diethyl succinate, and citronellol, were found at significantly higher levels, indicating a stabilization or reduction in the synthesis of most volatile compounds during the final stage. When *T. delbrueckii* was used in its immobilized form, a partially similar trend was observed. At 8 days, increases in nerol and β-damascenone mirrored those found in the free-cell condition, while at day 21, β-phenylethanol and acetaldehyde also showed comparable increases. However, by the end of the refermentation, only diethyl succinate remained significantly elevated.

In general, a significant increase in most volatile compounds was observed between days 8 and 21 of refermentation, highlighting this period as a critical window for the formation of key aroma constituents. A similar trend was found in sparkling wines produced with *L. thermotolerans* in free-cell inoculum, where a progressive and marked increase in volatile compounds during the early and middle stages of refermentation was seen. Conversely, *L. thermotolerans* in immobilized form showed a volatile compound production more variable and less predictable across the fermentation timeline. Despite this variability, sparkling wines produced using immobilized *L. thermotolerans* cells exhibited a generally higher concentration of major volatile compounds compared to their counterparts fermented with free cells. This suggests that, although kinetics may differ, the use of immobilized cells can influence enhancing the aromatic intensity and complexity of the final product.

**Figure 3:**
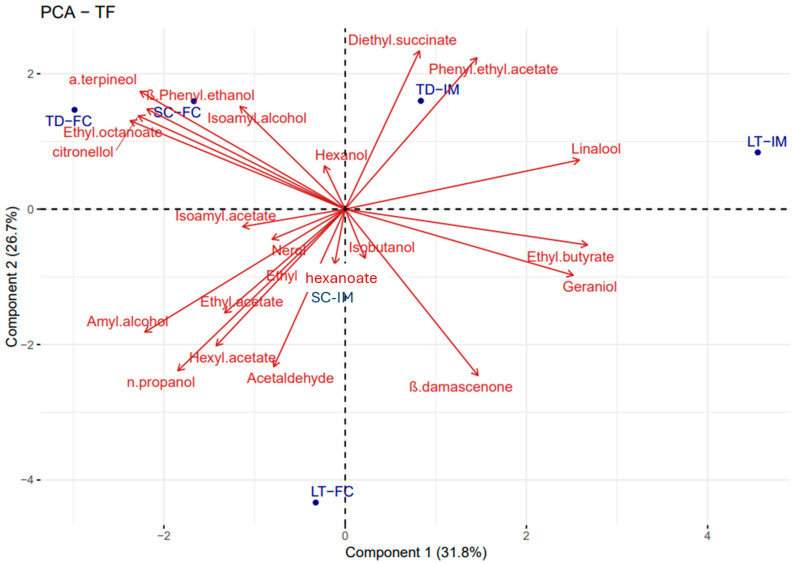
Principal component analysis of the volatile compounds in the sparkling wines obtained by the *S. cerevisiae* DiSVA 554, *T. delbrueckii* DiSVA 130, and *L. thermotolerans* DiSVA 322, free (FC) and immobilized cells (IM). The variance explained by PCA is PC 1 = 31.8% *x*-axis and PC 2 = 26.7% *y*-axis.

The results concerning the main volatile compounds detected at the end of the refermentation process were further analyzed by Principal Component Analysis (PCA), as shown in Figure 3.

This multivariate statistical approach was employed to evaluate the overall impact of the two different fermentation strategies, free versus immobilized yeast cells, on the final aromatic profile of the sparkling wines. The PCA biplot clearly revealed that the cell form used during the refermentation phase had a notable influence on the volatile composition of the wines. The distribution of the samples within the PCA space indicated a clear separation based on inoculum strategy: *T. delbrueckii* and *L. thermotolerans* free cells on the left quadrant and immobilized cells on the right, while *S. cerevisiae* free cells were in the upper left quadrant and immobilized cells were found in the lower left quadrant, suggesting that the physical state of the yeast significantly modulates the production of volatile aroma compounds. Moreover, separation due to the strain influence was also seen except for *S.* *cerevisiae* and *T. delbrueckii* that were placed in the upper left quadrant. This spatial distribution highlights that not only the yeast species but also its physical form during refermentation plays a relevant role in shaping the volatile composition and, consequently, the sensory identity of the final sparkling wine. Overall, the PCA highlighted the discriminative power of fermentation strategy in driving the aromatic profile of sparkling wines, supporting the hypothesis that yeast immobilization may serve as a technological tool to modulate wine aroma in a targeted and reproducible manner.

### 3.4. Sensorial Analysis

**Figure 4:**
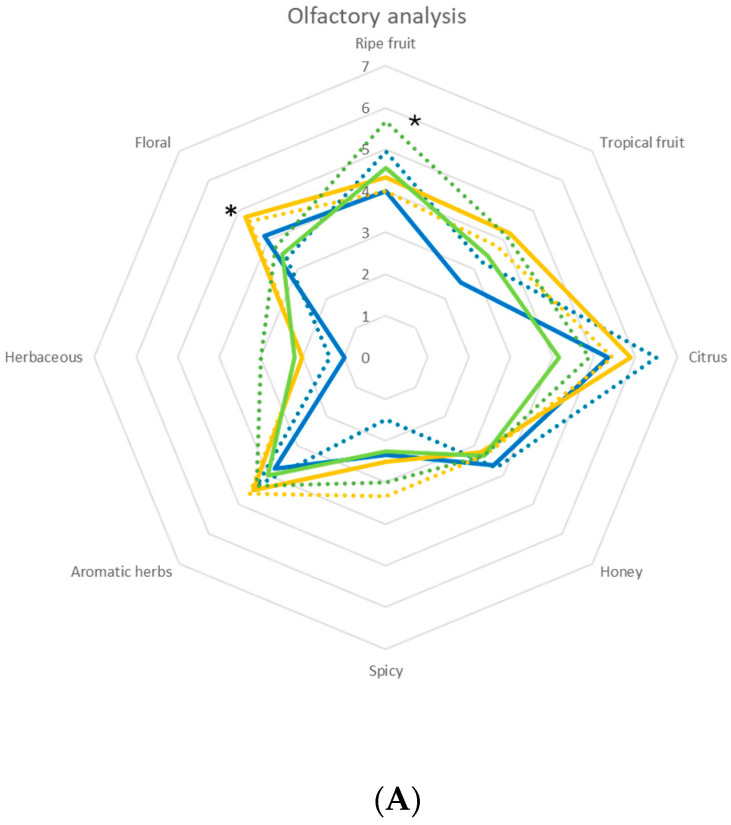

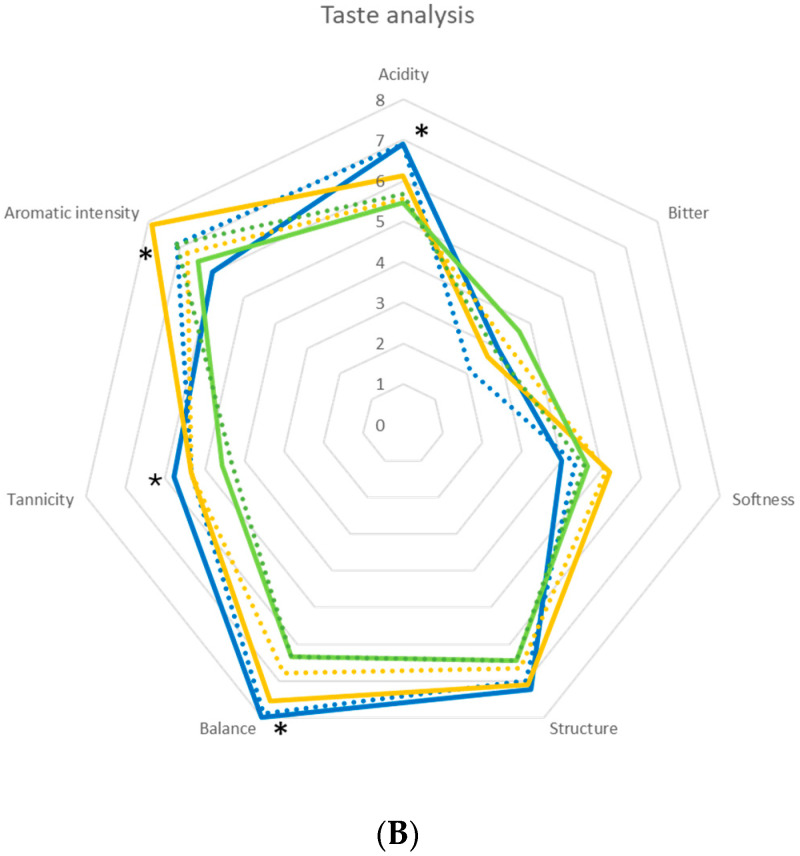
Sensory analysis of the sparkling wines obtained by free and immobilized cells. (**A**) Olfactory analysis. (**B**) Taste analysis: *S. cerevisiae* DiSVA 554, free (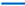) and immobilized (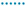); *T. delbrueckii* DiSVA, 130 free (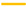) and immobilized (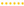); *L. thermotolerans* DiSVA 322, free (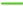) and immobilized (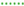). Note: * significantly different (Fisher ANOVA; *p*-value 0.05).

The sparkling wines were subjected to both olfactory and taste analyses after 6 months of bottle fermentation, and the results are reported in Figure 4.

Overall, all the sparkling wine samples were positively evaluated by the sensory panel and did not exhibit any organoleptic defects, confirming the suitability of the employed yeast strains and fermentation strategy for high-quality sparkling wine production. On the olfactory analysis (Figure 4A), relevant differences emerged depending on the yeast strain and the cell inoculation modality (free or immobilized). In particular, the sparkling wine produced with *L. thermotolerans* in its immobilized form showed a significant enhancement in the perception of ripe fruit aromas, suggesting a potential role of this yeast in enriching the fruity aromatic profile of the final product. On the other hand, sparkling wines fermented with *T. delbrueckii*, both in free and immobilized forms, were characterized by a significant increase in floral descriptors, indicating that this non-*Saccharomyces* yeast can positively influence the aromatic complexity of sparkling wines. Additionally, the use of *T. delbrueckii* in the free-cell form led to a noticeable intensification of overall aromatic intensity, highlighting its potential to boost the sensory impact of the sparkling wine. In terms of taste (Figure 4B), sparkling wines re-fermented with *S. cerevisiae* stood out for their higher perceived acidity and better balance between structure and freshness, confirming the well-established enological performance of this species. Notably, a general trend emerged in favor of immobilized yeast cells. Sparkling wines produced with immobilized cells achieved higher scores across most sensory descriptors compared to those obtained with free cells. This suggests that cell immobilization may play a positive role in modulating the metabolic activity of yeasts during secondary fermentation, ultimately enhancing the sensory quality and complexity of the final product.

## 4. Discussion

A wide variety of *S. cerevisiae* strains are commercially available for both the first and second fermentation of sparkling wine production. In recent years, there has been growing interest in indigenous *S. cerevisiae* and non-*Saccharomyces* yeasts to enhance the sensory properties of sparkling wines [20,21,22]. The use of native yeast starters in the production of base and sparkling wines can contribute to the typicality of the final product and may represent a valuable technological alternative to commercial starters [21,23]. In this context, autochthonous *S. cerevisiae* strains have been identified and proposed for use in both the first and second fermentations to help define the distinctive characteristics of sparkling wines [21,22,23,24]. Similarly, non-*Saccharomyces* yeasts have also been investigated for their potential to diversify and enhance the aromatic and sensory profiles of sparkling wines during both fermentation stages [25,26]. Indeed, different studies investigate the use of non-*Saccharomyces* in the production of base wine, but few research studies focus the attention on the use of non-conventional yeasts in secondary fermentation.

To produce base wine, *M. pulcherrima* and *T. delbrueckii* have been evaluated for their oenological potential. *M. pulcherrima* was shown to enhance foam persistence and contribute to the aromatic profile with distinctive smoky and floral notes [27]. In contrast, *T. delbrueckii* increased glycerol content, reduced volatile acidity, and improved foaming properties [16]. Only a few studies have investigated the use of non-*Saccharomyces* yeasts in secondary fermentation. *S. ludwigii* and *S. pombe* were evaluated by Ivit and Kamp [15], while *T. delbrueckii* strains showed promising results in studies by Canonico et al. [14] and Tofalo et al. [28]. Positive outcomes were also reported for *Starmerella bacillaris*, although it was unable to complete the secondary fermentation when used in pure culture [28].

In the present study, two previously selected non-*Saccharomyces* strains, *T. delbrueckii* DiSVA 130 and *L. thermotolerans* DiSVA 322, were evaluated during the secondary fermentation of sparkling wine, both as free and immobilized forms. The efficiency and effectiveness of the immobilizing yeast method to produce sparkling wines had already been examined in the past [28,29], but in *S. cerevisiae* models. Here, the influence of both non-*Saccharomyces* strains and the immobilization approach was monitored during secondary fermentation. Immobilization appeared to negatively affect the refermentation evolution of non-*Saccharomyces* strains, likely due to insufficient acclimatization and reduced resistance to stress factors such as ethanol, pressure, and nutrient limitation. Similar limitations have been previously reported for non-*Saccharomyces* yeasts immobilized in alginate or biological carriers, which showed slower sugar consumption and reduced fermentation efficiency compared to *S. cerevisiae* [29,30]. In contrast, *S. cerevisiae*, which is more tolerant to stressful winemaking conditions, did not exhibit these drawbacks and showed improved refermentation performance under immobilized conditions, as also observed by Puig-Pujol et al. [31] and Fumi et al. [32]. Despite the slower fermentation, the final ethanol concentration and residual sugars in wines produced with immobilized non-*Saccharomyces* strains remained comparable to those from free-cell fermentations, with only a slight increase in sugar (approximately 3 g/L). Notably, the secondary fermentation with immobilized yeasts yielded sparkling wines with enhanced volatile compound production and a more differentiated aromatic profile compared to free-cell trials. These findings align with previous reports on immobilized systems, which demonstrated an increase in esters, acids, and higher alcohols that contribute positively to wine aroma complexity [31,33].

Sensory analysis further confirmed these results, as immobilized trials obtained the highest scores in several of the evaluated descriptors, indicating the promising potential of *T. delbrueckii* DiSVA 130 and *L. thermotolerans* DiSVA 322 for enhancing the sensory attributes of sparkling wines. These findings demonstrate that yeast immobilization can modulate fermentation dynamics and aroma development, offering a promising strategy to tailor sparkling wine quality and sensory complexity.

This could be attributed to the alteration of the microenvironment and mass transfer dynamics associated with cell immobilization, which may promote prolonged metabolic activity. These results highlight the potential of immobilized *S. cerevisiae* to support and modulate the production of desirable aroma compounds during sparkling wine production. By promoting a more gradual and prolonged release of volatile substances, immobilization may allow greater control over the sensory development of the wine, with the potential to impart more complex and balanced aromatic profiles to the final product. This approach could therefore represent a promising strategy to improve the overall quality and differentiation of classic method sparkling wines. This suggests that immobilization may alter the dynamics of volatile compound production, potentially slowing or modulating the yeast’s metabolic activity over time.

Further studies are required to elucidate the behavior of immobilized non-*Saccharomyces* yeasts during sparkling wine production, with particular emphasis on their metabolic activity beyond mere fermentation performance.

## 5. Conclusions

The use of native *S. cerevisiae* strains and non-*Saccharomyces* yeasts offers new opportunities to define and enrich the aroma profile of sparkling wines. In the secondary fermentations, some selected strains of non-*Saccharomyces* strains show a promising potential in sparkling wine bottle fermentation. The yeast inoculum using the immobilization procedure may enhance aromatic complexity through acetate esters and higher alcohols. Sensory profiles, supported by sensory analyses, suggest immobilization to modulate aroma development over time. However, immobilized processes for non-*Saccharomyces* wine yeasts require close attention to acclimatization and greater management of stress factors (such as ethanol, pressure, and nutrients). For this, further studies are needed to understand the physiology and metabolism of immobilized non-*Saccharomyces* yeasts during production. The use of non-*Saccharomyces* strains and immobilization systems could represent a promising strategy to improve the quality and differentiation of traditional-method sparkling wines.

## Figures and Tables

**Table 1:** The main analytical characters of sparkling wines obtained by free and immobilized cells at the end of refermentation phase.

**Free Cells**	**Ethanol** **(%** ***v*** **/** ***v*** **)**	**Total Acidity** **(as Tartaric Acid g/L)**	**pH**	**Volatile Acidity** **(as Acetic Acid g/L)**	**Sugar Content** **(g/L)**
***S. cerevisiae***	11.92 ± 0.00 ^a^	4.36 ± 0.00 ^c^	3.57 ± 0.00 ^a^	0.49 ± 0.00 ^c^	1.04 ± 0.00 ^c^
***T. delbrueckii***	11.92 ± 0.00 ^a^	4.66 ± 0.00 ^b^	3.51 ± 0.00 ^c^	0.51 ± 0.00 ^b^	1.25 ± 0.00 ^b^
***L. thermotolerans***	11.90 ± 0.00 ^a^	4.61 ± 0.00 ^b^	3.53 ± 0.00 ^b^	0.51 ± 0.00 ^b^	1.33 ± 0.00 ^b^
**Immobilized Cells**	**Ethanol** **(%** ***v*** **/** ***v*** **)**	**Total Acidity** **(as Tartaric Acid g/L)**	**pH**	**Volatile Acidity** **(as Acetic Acid g/L)**	**Sugar Content** **(g/L)**
***S. cerevisiae***	11.91 ± 0.02 ^a^	5.68 ± 0.03 ^a^	3.42 ± 0.01 ^d^	0.42 ± 0.00 ^d^	1.32 ± 0.00 ^b^
***T. delbrueckii***	11.99 ± 0.03 ^a^	4.37 ± 0.04 ^c^	3.52 ± 0.00 ^b^^c^	0.54 ± 0.01 ^a^	3.38 ± 0.02 ^a^
***L. thermotolerans***	11.98 ± 0.02 ^a^	4.31 ± 0.04 ^c^	3.52 ± 0.01 ^b^	0.54 ± 0.01 ^a^	3.45 ± 0.08 ^a^

Data are means ± standard deviations. Data with different superscript letters (^a^, ^b^, ^c^, ^d^) within each column are significantly different (Duncan tests; *p* < 0.05).

**Table 2:** The main volatile compounds during the refermentation phase in free and immobilized form.

Free Cells	*S. cerevisiae* T8	*S. cerevisiae* T21	*S. cerevisiae* TF
**Esters (mg/L)**			
Ethyl butyrate	0.133 ± 0.017 ^a^	0.062 ± 0.007 ^b^	0.042 ± 0.018 ^b^
Ethyl acetate	18.06 ± 2.30 ^b^	22.64 ± 0.12 ^a^	11.57 ± 0.20 ^c^
Phenyl ethyl acetate	0.044 ± 0.004 ^a^	0.014 ± 0.002 ^b^	0.038 ± 0.003 ^a^
Ethyl hexanoate	0.062 ± 0.004 ^b^	0.096 ± 0.003 ^a^	0.018 ± 0.000 ^c^
Ethyl octanoate	0.017 ± 0.001 ^a^	0.007 ± 0.001 ^b^	0.005 ± 0.000 ^b^
Isoamyl acetate	15.97 ± 0.06 ^b^	17.94 ± 0.40 ^a^	14.73 ± 0.15 ^c^
Hexyl acetate	0.002 ± 0.001 ^a^	0.001 ± 0.000 ^a^	0.003 ± 0.001 ^a^
Diethyl succinate	0.004 ± 0.000 ^a^	0.008 ± 0.000 ^a^	0.016 ± 0.000 ^a^
**Alcohols (mg/L)**			
n-Propanol	28.64 ± 1.24 ^a^	29.50 ± 0.42 ^a^	22.43 ± 0.23 ^b^
Isobutanol	7.43 ± 0.16 ^b^	10.79 ± 0.01 ^a^	4.65 ± 0.26 ^c^
Amyl alcohol	3.97 ± 0.11 ^b^	6.25 ± 0.44 ^a^	3.03 ± 0.90 ^b^
Isoamylic alcohol	79.40 ± 4.70 ^a^	80.39 ± 0.035 ^a^	63.61 ± 0.10 ^b^
*β*-Phenyl ethanol	60.25 ± 0.13 ^b^	73.33 ± 0.26 ^a^	63.12 ± 0.29 ^b^
Hexanol	0.050 ± 0.016 ^b^	0.095 ± 0.006 ^a^	0.029 ± 0.002 ^b^
**Carbonyl compounds(mg/L)**			
Acetaldehyde	41.13 ± 0.60 ^a^	14.38 ± 0.10 ^b^	1.40 ± 0.53 ^c^
Monoterpenes			
Linalool	0.020 ± 0.013 ^ab^	0.043 ± 0.003 ^a^	0.013 ± 0.001 ^b^
Geraniol	0.023 ± 0.002 ^a^	0.002 ± 0.000 ^b^	0.004 ± 0.002 ^b^
Nerol	0.014 ± 0.008 ^a^	0.007 ± 0.000 ^a^	0.006 ± 0.000 ^a^
*α*-terpineol	0.071 ± 0.045 ^a^	0.000 ± 0.000 ^a^	0.080 ± 0.001 ^a^
Citronellol	0.026 ± 0.001 ^b^	0.030 ± 0.008 ^b^	0.088 ± 0.001 ^a^
**Norisoprenoids (mg/L)**			
*β*-damascenone	0.006 ± 0.001 ^a^	0.003 ± 0.000 ^b^	0.003 ± 0.000 ^b^
**Immobilized Cells**	***S. cerevisiae*** **T8**	***S. cerevisiae*** **T21**	***S. cerevisiae*** **TF**
**Esters (mg/L)**			
Ethyl butyrate	0.013 ± 0.000 ^a^	0.020 ± 0.007 ^a^	0.067 ± 0.047 ^a^
Ethyl acetate	5.59 ± 0.65 ^b^	11.47 ± 1.58 ^a^	14.42 ± 0.28 ^a^
Phenyl ethyl acetate	0.013 ± 0.002 ^b^	0.055 ± 0.007 ^a^	0.020 ± 0.000 ^b^
Ethyl hexanoate	0.199 ± 0.196 ^a^	0.228 ± 0.017 ^a^	0.132 ± 0.004 ^a^
Ethyl octanoate	0.004 ± 0.000 ^a^	0.006 ± 0.001 ^a^	0.005 ± 0.000 ^a^
Isoamyl acetate	5.71 ± 1.21 ^b^	7.82 ± 0.20 ^ab^	10.14 ± 0.38 ^a^
Hexyl acetate	0.002 ± 0.000 ^a^	0.001 ± 0.000 ^a^	0.002 ± 0.000 ^a^
Diethyl succinate	0.022 ± 0.001 ^a^	0.018 ± 0.002 ^ab^	0.015 ± 0.000 ^b^
**Alcohols (mg/L)**			
n-Propanol	25.42 ± 1.69 ^a^	24.94 ± 0.23 ^a^	23.20 ± 0.67 ^a^
Isobutanol	6.16 ± 0.08 ^b^	7.71 ± 0.11 ^a^	7.67 ± 0.16 ^a^
Amyl alcohol	15.61 ± 1.19 ^a^	13.55 ± 1.13 ^a^	4.12 ± 0.10 ^b^
Isoamylic alcohol	52.58 ± 4.42 ^b^	36.55 ± 0.28 ^c^	67.53 ± 0.63 ^a^
*β*-Phenyl ethanol	72.50 ± 0.73 ^a^	59.54 ± 0.54 ^a^	59.25 ± 0.48 ^a^
Hexanol	0.004 ± 0.001 ^c^	0.051 ± 0.003 ^b^	0.067 ± 0.001 ^a^
**Carbonyl compounds (mg/L)**			
Acetaldehyde	4.44 ± 0.23 ^b^	8.39 ± 0.13 ^a^	1.80 ± 0.09 ^c^
**Monoterpenes**			
Linalool	0.023 ± 0.007 ^a^	0.007 ± 0.000 ^b^	0.005 ± 0.000 ^b^
Geraniol	0.006 ± 0.001 ^b^	0.019 ± 0.001 ^a^	0.017 ± 0.002 ^a^
Nerol	0.024 ± 0.002 ^a^	0.007 ± 0.000 ^b^	0.004 ± 0.000 ^b^
*α*-terpineol	0.064 ± 0.007 ^a^	0.015 ± 0.003 ^b^	0.029 ± 0.012 ^b^
Citronellol	0.015 ± 0.003 ^ab^	0.015 ± 0.001 ^b^	0.050 ± 0.019 ^a^
**Norisoprenoids** **(mg/L)**			
*β*-damascenone	0.005 ± 0.000 ^b^	0.013 ± 0.002 ^a^	0.010 ± 0.001 ^a^
**Free Cells**	***T. delbrueckii*** **T8**	***T. delbrueckii*** **T21**	***T. delbrueckii*** **TF**
**Esters (mg/L)**			
Ethyl butyrate	0.105 ± 0.043 ^a^	0.021 ± 0.001 ^b^	0.000 ± 0.000 ^b^
Ethyl acetate	19.29 ± 0.12 ^c^	23.59 ± 0.24 ^a^	20.54 ± 0.60 ^b^
Phenyl ethyl acetate	0.040 ± 0.002 ^b^	0.056 ± 0.001 ^a^	0.023 ± 0.006 ^c^
Ethyl hexanoate	0.049 ± 0.039 ^a^	0.022 ± 0.002 ^a^	0.010 ± 0.001 ^a^
Ethyl octanoate	0.001 ± 0.000 ^c^	0.003 ± 0.000 ^b^	0.006 ± 0.001 ^a^
Isoamyl acetate	25.65 ± 0.31 ^a^	18.83 ± 0.16 ^b^	8.79 ± 0.30 ^c^
Hexyl acetate	0.003 ± 0.000 ^a^	0.001 ± 0.000 ^a^	0.002 ± 0.000 ^a^
Diethyl succinate	0.003 ± 0.000 ^c^	0.009 ± 0.003 ^b^	0.017 ± 0.001 ^a^
**Alcohols (mg/L)**			
n-Propanol	21.85 ± 0.51 ^b^	27.83 ± 0.42 ^a^	22.52 ± 0.08 ^b^
Isobutanol	7.37 ± 0.26 ^a^	5.53 ± 0.37 ^b^	4.26 ± 0.23 ^c^
Amyl alcohol	4.34 ± 2.28 ^a^	4.08 ± 0.01 ^a^	3.77 ± 0.32 ^a^
Isoamylic alcohol	53.63 ± 0.36 ^c^	79.38 ± 0.56 ^a^	64.12 ± 0.59 ^b^
*β*-Phenyl ethanol	80.34 ± 0.00 ^b^	91.68 ± 0.00 ^a^	69.27 ± 0.00 ^c^
Hexanol	0.020 ± 0.011 ^b^	0.026 ± 0.008 ^b^	0.078 ± 0.001 ^a^
**Carbonyl** **Compounds (mg/L)**			
Acetaldehyde	31.37 ± 0.53 ^b^	93.78 ± 0.42 ^a^	3.10 ± 0.59 ^c^
Monoterpens			
Linalool	0.036 ± 0.003 ^a^	0.007 ± 0.000 ^c^	0.014 ± 0.001 ^b^
Geraniol	0.030 ± 0.000 ^a^	0.023 ± 0.001 ^b^	0.000 ± 0.000 ^c^
Nerol	0.055 ± 0.004 ^a^	0.027 ± 0.001 ^b^	0.000 ± 0.000 ^c^
*α*-terpineol	0.051 ± 0.001 ^b^	0.091 ± 0.010 ^a^	0.063 ± 0.013 ^ab^
Citronellol	0.025 ± 0.004 ^c^	0.068 ± 0.012 ^b^	0.132 ± 0.011 ^a^
**Norisoprenoids (mg/L)**			
*β*-damascenone	0.021 ± 0.001 ^a^	0.010 ± 0.001 ^b^	0.003 ± 0.000 ^c^
**Immobilized Cells**	***T. delbrueckii*** **T8**	***T. delbrueckii*** **T21**	***T. delbrueckii*** **TF**
**Esters (mg/L)**			
Ethyl butyrate	0.024 ± 0.001 ^b^	0.089 ± 0.003 ^a^	0.016 ± 0.022 ^b^
Ethyl acetate	21.73 ± 1.26 ^a^	18.30 ± 0.80 ^b^	12.73 ± 0.25 ^c^
Phenyl ethyl acetate	0.078 ± 0.001 ^a^	0.053 ± 0.002 ^b^	0.030 ± 0.002 ^c^
Ethyl hexanoate	0.104 ± 0.055 ^b^	0.167 ± 0.012 ^a^	0.018 ± 0.001 ^c^
Ethyl octanoate	0.007 ± 0.001 ^a^	0.004 ± 0.001 ^ab^	0.003 ± 0.000 ^b^
Isoamyl acetate	4.53 ± 0.00 ^c^	7.03 ± 0.13 ^b^	10.80 ± 0.51 ^a^
Hexyl acetate	0.001 ± 0.000 ^a^	0.001 ± 0.000 ^a^	0.000 ± 0.000 ^b^
Diethyl succinate	0.001 ± 0.000 ^c^	0.017 ± 0.002 ^b^	0.022 ± 0.003 ^a^
**Alcohols (mg/L)**			
n-Propanol	29.22 ± 1.12 ^a^	23.10 ± 0.13 ^b^	19.04 ± 0.47 ^c^
Isobutanol	10.47 ± 0.30 ^b^	11.37 ± 0.26 ^a^	4.97 ± 0.11 ^c^
Amyl alcohol	4.16 ± 0.37 ^b^	12.70 ± 2.69 ^a^	3.05 ± 0.25 ^b^
Isoamylic alcohol	75.96 ± 0.06 ^a^	46.30 ± 0.42 ^c^	69.33 ± 0.70 ^b^
*β*-Phenyl ethanol	67.86 ± 0.00 ^b^	69.27 ± 0.00 ^a^	59.06 ± 0.00 ^c^
Hexanol	0.033 ± 0.007 ^a^	0.042 ± 0.007 ^a^	0.037 ± 0.004 ^a^
**Carbonyl** **Compounds (mg/L)**			
Acetaldehyde	1.90 ± 0.14 ^c^	28.14 ± 0.04 ^a^	3.77 ± 3.58 ^b^
Monoterpenes			
Linalool	0.016 ± 0.006 ^a^	0.007 ± 0.002 ^a^	0.015 ± 0.000 ^a^
Geraniol	0.007 ± 0.001 ^b^	0.017 ± 0.003 ^a^	0.021 ± 0.001 ^a^
Nerol	0.011 ± 0.000 ^a^	0.005 ± 0.001 ^b^	0.004 ± 0.000 ^b^
*α*-terpineol	0.055 ± 0.006 ^a^	0.052 ± 0.007 ^ab^	0.032 ± 0.008 ^b^
Citronellol	0.026 ± 0.016 ^a^	0.055 ± 0.010 ^a^	0.063 ± 0.011 ^a^
**Norisoprenoids (mg/L)**			
*β*-damascenone	0.010 ± 0.000 ^a^	0.011 ± 0.000 ^a^	0.003 ± 0.000 ^b^
**Free Cells**	***L. thermotolerans*** **T8**	***L. thermotolerans*** **T21**	***L. thermotolerans*** **TF**
**Esters (mg/L)**			
Ethyl butyrate	0.000 ± 0.000 ^b^	0.034 ± 0.010 ^b^	0.225 ± 0.017 ^a^
Ethyl acetate	24.68 ± 0.22 ^a^	17.84 ± 0.12 ^c^	19.89 ± 0.06 ^b^
Phenyl ethyl acetate	0.037 ± 0.001 ^b^	0.065 ± 0.001 ^a^	0.015 ± 0.002 ^c^
Ethyl hexanoate	0.011 ± 0.001 ^b^	0.023 ± 0.001 ^a^	0.019 ± 0.001 ^a^
Ethyl octanoate	0.005 ± 0.001 ^a^	0.006 ± 0.000 ^a^	0.002 ± 0.000 ^b^
Isoamyl acetate	22.69 ± 1.56 ^a^	17.46 ± 0.30 ^b^	11.84 ± 0.29 ^c^
Hexyl acetate	0.003 ± 0.000 ^a^	0.001 ± 0.000 ^b^	0.004 ± 0.000 ^a^
Diethyl succinate	0.015 ± 0.001 ^a^	0.007 ± 0.001 ^c^	0.011 ± 0.000 ^b^
**Alcohols (mg/L)**			
n-Propanol	24.92 ± 0.32 ^b^	27.92 ± 0.86 ^a^	26.72 ± 0.21 ^a^
Isobutanol	5.51 ± 1.34 ^a^	5.94 ± 0.87 ^a^	4.67 ± 0.03 ^a^
Amyl alcohol	4.43 ± 0.11 ^a^	3.91 ± 0.52 ^a^	4.18 ± 0.11 ^a^
Isoamylic alcohol	84.78 ± 0.23 ^a^	79.95 ± 0.34 ^b^	56.13 ± 0.88 ^c^
*β*-Phenyl ethanol	76.69 ± 0.00 ^a^	78.54 ± 0.48 ^a^	57.96 ± 0.17 ^b^
Hexanol	0.040 ± 0.002 ^b^	0.078 ± 0.015 ^a^	0.032 ± 0.000 ^b^
**Carbonyl** **Compounds (mg/L)**			
Acetaldehyde	94.56 ± 2.97 ^a^	56.71 ± 0.03 ^b^	5.99 ± 0.58 ^c^
Monoterpenes			
Linalool	0.011 ± 0.000 ^a^	0.006 ± 0.000 ^a^	0.009 ± 0.006 ^a^
Geraniol	0.029 ± 0.001 ^a^	0.002 ± 0.000 ^c^	0.017 ± 0.005 ^b^
Nerol	0.005 ± 0.001 ^a^	0.003 ± 0.000 ^a^	0.004 ± 0.001 ^a^
*α*-terpineol	0.018 ± 0.006 ^a^	0.040 ± 0.025 ^a^	0.011 ± 0.008 ^a^
Citronellol	0.062 ± 0.023 ^a^	0.057 ± 0.032 ^a^	0.055 ± 0.021 ^a^
**Norisoprenoids (mg/L)**			
*β*-damascenone	0.005 ± 0.001 ^b^	0.005 ± 0.000 ^b^	0.013 ± 0.001 ^a^
**Immobilized Cells**	***L. thermotolerans*** **T8**	***L. thermotolerans*** **T21**	***L. thermotolerans*** **TF**
**Esters (mg/L)**			
Ethyl butyrate	0.005 ± 0.000 ^b^	0.007 ± 0.001 ^b^	0.568 ± 0.007 ^a^
Ethyl acetate	11.08 ± 1.09 ^b^	14.53 ± 0.02 ^a^	13.18 ± 0.23 ^a^
Phenyl ethyl acetate	0.022 ± 0.001 ^b^	0.048 ± 0.006 ^a^	0.040 ± 0.002 ^a^
Ethyl hexanoate	0.107 ± 0.005 ^b^	0.198 ± 0.021 ^a^	0.018 ± 0.000 ^c^
Ethyl octanoate	0.005 ± 0.000 ^a^	0.002 ± 0.000 ^b^	0.002 ± 0.000 ^b^
Isoamyl acetate	6.82 ± 0.27 ^b^	9.98 ± 0.21 ^a^	8.53 ± 1.66 ^ab^
Hexyl acetate	0.001 ± 0.000 ^a^	0.001 ± 0.000 ^a^	0.001 ± 0.000 ^a^
Diethyl succinate	0.002 ± 0.000 ^b^	0.018 ± 0.002 ^a^	0.018 ± 0.003 ^a^
**Alcohols (mg/L)**			
n-Propanol	25.64 ± 0.46 ^a^	16.80 ± 0.18 ^b^	18.44 ± 1.72 ^b^
Isobutanol	7.69 ± 0.33 ^a^	5.37 ± 0.22 ^b^	4.45 ± 0.59 ^b^
Amyl alcohol	3.57 ± 0.32 ^a^	3.46 ± 0.40 ^a^	1.92 ± 1.04 ^a^
Isoamylic alcohol	61.58 ± 0.32 ^b^	67.42 ± 0.13 ^a^	56.75 ± 0.14 ^c^
*β*-Phenyl ethanol	54.78 ± 0.52 ^a^	65.51 ± 0.57 ^a^	58.22 ± 0.16 ^a^
Hexanol	0.014 ± 0.000 ^b^	0.017 ± 0.001 ^b^	0.059 ± 0.004 ^a^
**Carbonyl** **Compounds (mg/L)**			
Acetaldehyde	2.26 ± 0.003 ^b^	35.72 ± 3.37 ^a^	1.18 ± 0.26 ^b^
Monoterpenes			
Linalool	0.029 ± 0.006 ^b^	0.033 ± 0.002 ^b^	0.103 ± 0.006 ^a^
Geraniol	0.003 ± 0.001 ^c^	0.016 ± 0.001 ^b^	0.022 ± 0.000 ^a^
Nerol	0.015 ± 0.002 ^a^	0.004 ± 0.001 ^b^	0.000 ± 0.000 ^c^
*α*-terpineol	0.035 ± 0.003 ^a^	0.036 ± 0.002 ^a^	0.007 ± 0.004 ^b^
Citronellol	0.023 ± 0.007 ^b^	0.045 ± 0.008 ^a^	0.046 ± 0.004 ^a^
**Norisoprenoids (mg/L)**			
*β*-damascenone	0.019 ± 0.002 ^a^	0.010 ± 0.001 ^b^	0.010 ± 0.001 ^b^

Data are means ± standard deviations. Data with different superscript letters (^a, b, c^) within each column are significantly different (Duncan tests; *p* < 0.05).

## Data Availability

The original contributions presented in the study are included in the article and Supplementary Materials, further inquiries can be directed to the corresponding author.

## Supplementary Materials

The following supporting information can be downloaded at: https://www.mdpi.com/article/10.3390/foods14173007/s1. Figure S1: Heatmap of the concentration of the main volatile compounds during different stages of secondary fermentation for each strain tested in free and immobilized form.
