# Supplementary material for: Free and Immobilized Cells of Torulaspora delbrueckii and Lachancea thermotolerans in Sparkling Wine: Innovative Application in Secondary Bottle Fermentation
Source: Foods. 2025 Aug 28;14(17):3007. doi: 10.3390/foods14173007 (PMC12428465; doi:10.3390/foods14173007)
Supplement: Supplementary file 1 [file foods-14-03007-s001.zip › foods-3831804-supplementary.pptx]

## Slide 1
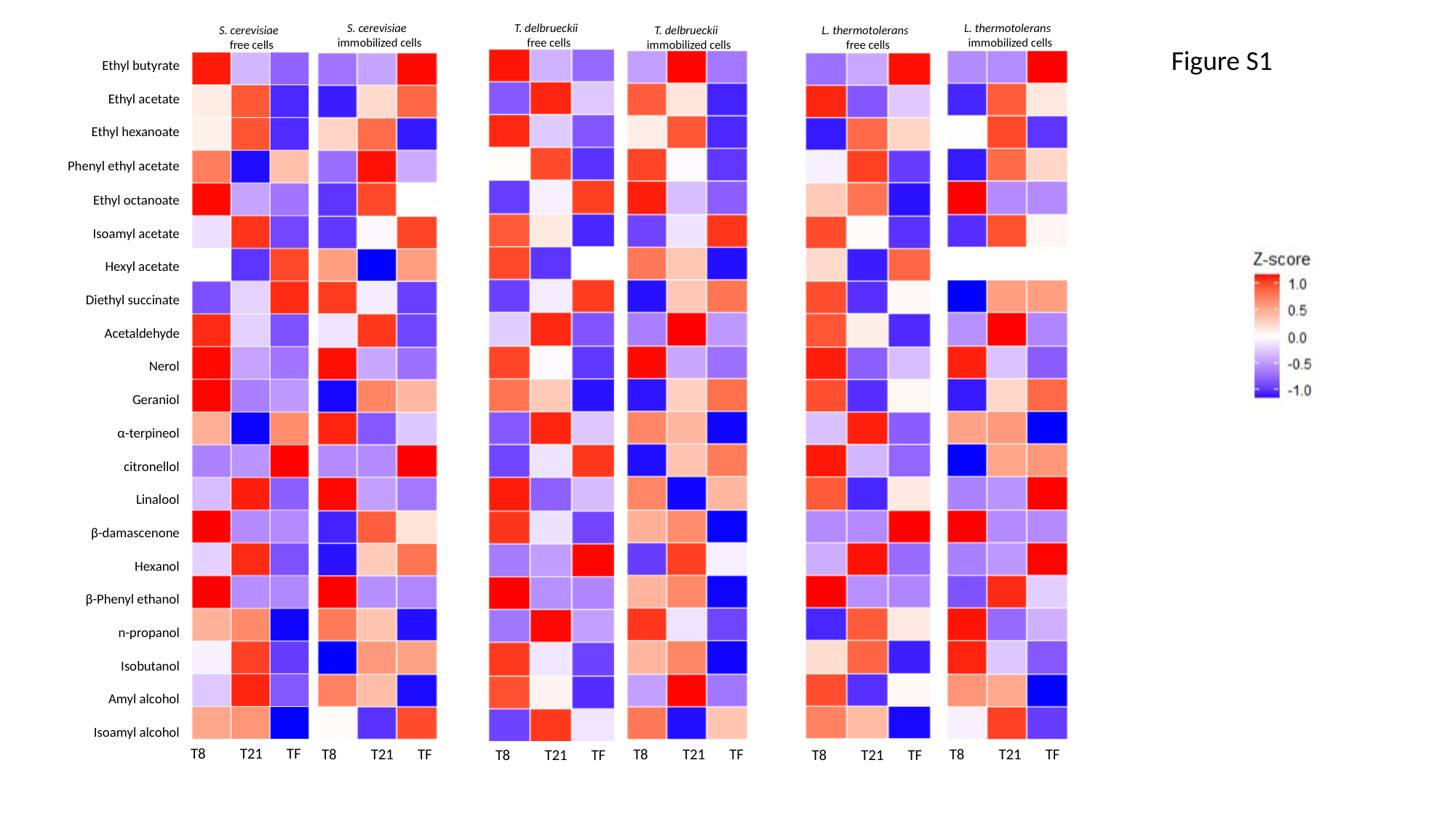

S. cerevisiae
immobilized cells
T. delbrueckii
free cells
L. thermotolerans
immobilized cells
S. cerevisiae
free cells
T. delbrueckii
immobilized cells
L. thermotolerans
free cells
Figure S1
| Ethyl butyrate |
| --- |
| Ethyl acetate |
| Ethyl hexanoate |
| Phenyl ethyl acetate |
| Ethyl octanoate |
| Isoamyl acetate |
| Hexyl acetate |
| Diethyl succinate |
| Acetaldehyde |
| Nerol |
| Geraniol |
| α-terpineol |
| citronellol |
| Linalool |
| β-damascenone |
| Hexanol |
| β-Phenyl ethanol |
| n-propanol |
| Isobutanol |
| Amyl alcohol |
| Isoamyl alcohol |
T8 T21 TF
T8 T21 TF
T8 T21 TF
T8 T21 TF
T8 T21 TF
T8 T21 TF
